# Mechanistic Approach on the Pulmonary Oxido-Inflammatory Stress Induced by Cobalt Ferrite Nanoparticles in Rats

**DOI:** 10.1007/s12011-023-03700-5

**Published:** 2023-05-16

**Authors:** Eman I. Hassanen, Rehab E. Abdelrahman, Hassan Aboul-Ella, Marwa A. Ibrahim, Samaa El-Dek, Mohamed Shaalan

**Affiliations:** 1 Department of Pathology, Faculty of Veterinary Medicine, Cairo University, P.O. Box 12211, Giza, Egypt; 2 Department of Toxicology and Forensic Medicine, Faculty of Veterinary Medicine, Cairo University, Giza, Egypt; 3 Department of Microbiology, Faculty of Veterinary Medicine, Cairo University, Giza, Egypt; 4 Department of Biochemistry and Molecular Biology, Faculty of Veterinary Medicine, Cairo University, Giza, Egypt; 5 Department of Material Science and Nanotechnology, Faculty of Postgraduate Studies for Advanced Sciences, Beni-Suef University, Beni-Suef, Egypt; 6 Polymer Institute, Slovak Academy of Science, Bratislava, Slovakia

**Keywords:** Gene expression, Inflammation, Magnetic nanoparticles, Oxidative stress, Pathology

## Abstract

Cobalt ferrite nanoparticles (CFN) are employed in data storage, imaging, medication administration, and catalysis due to their superparamagnetic characteristics. The widespread use of CFN led to significantly increased exposure to people and the environment to these nanoparticles. Until now, there is not any published paper describing the adverse effect of repeated oral intake of this nanoformulation on rats’ lungs. So, the current research aims to elucidate the pulmonary toxicity prompted by different concentrations of CFN in rats as well as to explore the mechanistic way of such toxicity. We used 28 rats that were divided equally into 4 groups. The control group received normal saline, and the experimental groups received CFN at dosage levels 0.05, 0.5, and 5 mg/kg bwt. Our findings revealed that CFN enhanced dose-dependent oxidative stress manifested by raising in the MDA levels and declining in the GSH content. The histopathological examination revealed interstitial pulmonary inflammation along with bronchial and alveolar damage in both 0.5 and 5 mg CFN given groups. All these lesions were confirmed by the immunohistochemical staining that demonstrated strong iNOS and Cox-2 protein expression. There was also a significant upregulation of TNF*α*, Cox-2, and IL-1*β* genes with downregulation of IL-10 and TGF-*β* genes. Additionally, the group receiving 0.05 mg CFN did not exhibit any considerable toxicity in all measurable parameters. We concluded that the daily oral intake of either 0.5 or 5 mg CFN, but not 0.05 mg, could induce pulmonary toxicity via NPs and/or its leached components (cobalt and iron)-mediated oxido-inflammatory stress. Our findings may help to clarify the mechanisms of pulmonary toxicity generated by these nanoparticles through outlining the standards for risk assessment in rats as a human model.

## Introduction

Cobalt ferrite nanoparticles (CNP) have received increasing attention due to their widespread therapeutic and agricultural applicability [1]. Additionally, the improved magnetic characteristics versus iron oxide nanoparticles are promising in nearly every area of biotechnology, including biosensors, separation and purification, drug administration, imaging, and treatment delivery systems [2]. CFN is one of the main categories of metal magnetic-engineered nanoparticle preparations [3]. With their unique characteristics and wide range of promising applications and incorporation in different medical, veterinary, and engineering fields, increased concerns have been raised about their capability to cause toxicity as well as their predicted public health possible adverse effects [4].

In spite of increasing the use of nanomaterials in various field, they are a double-edged sword for future medicine because of their unfavorable consequences on the exposed manufacturers, industry workers, and patients [5, 6]. Because of their higher chemical reactivity and biological activity, it was widely believed that nanoparticles would be more toxic than larger particles [7]. CFN can enter the body by several ways mainly inhalation and ingestion [8], and cross cell membranes then interact with subcellular elements causing cell membrane disruption and cell death [9]. Several in vitro studies revealed the cytotoxic effect of CFN [10, 11], but the toxicity of CFN showed various results in different experimental animal models with lack of the potential mechanism especially from the pathological and molecular insights [12–14]. There are several factors affecting CFN toxicity in the biological system including coating material, size, concentration, route of administration, frequency of exposure, and animal model [15]. CFN had the potential to pass across tissue barriers and move through the blood to other organs especially lungs, spleen, liver, and kidneys, causing oxidative stress damage and inflammatory responses [16]. Recent study confirmed that short-term inhalation of CFN could induce respiratory toxicity in Guinea pig [17]. The alveoli are not as well shielded from NPs because of their vast surface area and close air-blood interaction [18]. So, lung is the primary target of NPs even when taken orally or by other routes as inhalation, making lung NPs-based toxicity one of the most noticeable and concerning affections [19]. Upon administration of CFN to pregnant albino rats, CFN crossed the blood-placenta barrier and deposited in the fetus organs, inducing oxidative damage [13]. The toxicity of CFN may be related to the particle itself or its leakage ions (cobalt and iron) which prompted reactive oxygen species (ROS) overgeneration resulting in lipid peroxidation, protein degradation, DNA damage leading to cell death [20].

The effects of nanoparticles on biological systems and the possibility of respiratory risks will depend on their various features, including size, shape, surface charge, chemical properties, solubility, and degree of agglomeration [21]. Recent research on the possible occupational and environmental impacts of NPs has shown that multifocal granulomas, peri-bronchial inflammation, progressive interstitial fibrosis, persistent inflammatory reactions, collagen deposition, and oxidative stress are some unfavorable respiratory consequences for NPs exposure [22]. Research directed toward this area either in the form of whole combined or individual fragments of metal-based NPs investigatory studies is the need of the hour. The current study’s main objective is to highlight and figure out the potential and actual respiratory toxic effect of CFN and/or its leached components (Co and Fe), one of the most prominent metal-based engineered magnetic nanoparticles, with clear and obvious research questions and gaps that should be covered and answered.

## Materials and Methods

### Preparation of Nanoparticles

CFN were synthesized via one-step combustion method according to Varma et al., [23] with some modification. It involved the reaction between all metal precursors in their nitrate form with the equimolar ratio of citric acid. Herein, 2 moles of iron nitrate (Fe (NO_3_)_3_ · 9H_2_O, Sigma-Aldrich, St Louis, MO, USA) were added to one mole of cobalt nitrate (Co (NO_3_)_2_ · 6H_2_O, Sigma-Aldrich, St Louis, MO, USA) and then dissolved on a magnetic stirrer thoroughly. In another beaker, the citric acid was dissolved in distilled H_2_O and added dropwise to the metal nitrate precursor solution. The pH value was thereafter adjusted using drops of ammonia solution till reaching neutrality. The viscous gel was formed after boiling. At this point, maximum energy will be released from the chemical reaction after being pasty. The frothy fluffy powder was obtained with blackish gray color. The collected powder was then dried in 120 °C to assure homogeneity of temperature. The nanoparticles were will grinded to a very fine powder using the agate mortar and sieved till being homogeneous.

### Characterization of the Synthesized Nanoparticles

X-ray diffraction analysis was carried out to confirm phase formation and crystalline phases existing in the sample using an X-ray diffractometer (analytical-x’ pertpro, Cuk_α1_ radiation, *λ* = 1.5404 Å, 45 kV, 40 mA, Netherlands). The results were collected in the 2-theta range 20° ≤ 2θ ≤ 70° with a step size =0.02° with an irradiation time of 0.5 s/ step. Additionally, the particle size was calculated using Scherrer equation [24]. Fourier transform infrared (FTIR) studies were performed in order to assure the phase purity and the nature of chemical bonds formed in CFN using FTIR (Thermo Fisher Scientific Inc., Pittsburgh, PA, USA). Furthermore, the mean hydrodynamic particle size measured by dynamic light scattering (DLS) and the zeta potential of CFN that correlated to the magnitude of the electrical charge at the particle surface and molecular weight of large polymeric substances dispersed in water were measured using Nano-Zetasizer 3000 HS (Malvern Instruments, Malvern, UK). Field emission scanning electron microscope was imaged for the powdered sample using Zeiss Sigma 500VP Analytical FE-SEM, Carl Zeiss (Germany) using accelerating voltage up to 30kV.

### Animals and Experimental Design

The Institutional Animal Care and Use Committee of Cairo University gave its approval to the experimental protocol, which was carried out in accordance with the European Council Directive (EU2010/63) requirements. Our investigation was done on 28 male Wistar rats with an average weight of 150–170 g. They were purchased from the Laboratory Animals’ unit in the Faculty of Veterinary Medicine, Cairo University, Egypt. They were kept in polymer cages covered by sawdust, as well as maintained at 22 °C, 55% relative humidity, and a 12-h light cycle every day. Rats were given dry commercial standard pellets (Al-Watania food Co., Giza, Egypt) to eat during the experiment, and they were given unlimited access to tap water. Prior to the trial, the rats were habituated for 2 weeks.

Rats were randomly assigned to four groups (*n* = 7). The therapies were given via oral route throughout the 14 days on daily basis, and each rat received 1 mL of the treatment daily. The negative control group (Group 1) got only normal saline, whereas, CFN were administered to Groups 2, 3, and 4 at doses of 0.05, 0.5, and 5 mg/kg BWT, sequentially. Although, there is no reference dose of CFN in rats, however, one recent study described the in vivo toxicity of this nanoformulation in mice **[**25**]**. Therefore, we used this study as a reference for choosing the high dose of NPs (5 mg) by using the dose conversion formula between different experimental animal species (https://dosecal.cftri.res.in/index.php). Furthermore, the low doses (0.5 and 0.05 mg) were also selected to explore the safe dosage level of CFN that can be used in medicine.

### Sampling

At 14 days postdosing, lungs were obtained from all rats in different groups. Portion of them was kept at −80 °C until used for molecular and biochemical analysis, while the remaining portions were fixed in 10% neutral buffered formalin for histopathological and immunohistochemical examination.

### Oxidative Stress Evaluations

The levels of malondialdehyde (MDA) and reduced glutathione (GSH) were estimated in the lung tissue of each group using the instructions of commercially available colorimetric kits (Biodiagnostic, Cairo, Egypt).

### Histopathological Examination

In order to generate paraffin-embedded tissue sections, formalin-fixed lung tissue samples were drained using graded ethanol, cleansed by Xylene, impregnated in paraffin wax, and sectioned at 4.5 μm. These sections were then stained with H&E and examined under a light Olympus microscope BX43 to examine their histological organization. After that, we captured photos utilizing an Olympus DP27 camera linked to CellSens dimensions software (Product Version, 1.13; Core Version, XV 3.12 (Build 13479)) (https://www.olympus-lifescience.com/en/software/cellsens/)[26].

Stained slides were provided as a blinded set to the veterinary pathologists (1, 2, 3, ...) and were semi-quantitatively scored and assigned on a scale (1–5) for each pathological parameter. We graded the severity and distribution of vascular damage, bronchial and bronchiolar damage, alveolar damage, airway inflammation, airway edema, hemorrhage, and interstitial inflammation, as follows: (1) normal histology, (2) slight < 10% tissue damage (TD), (3) mild 11–25% TD, (4) moderate 26–50% TD, and (5) severe > 50% TD **[**27**]**.

### Immunohistochemical Staining

Avidin-biotin-peroxidase complex (ABC) was used in an immunohistochemical analysis to identify iNOS and Cox-2 as inflammatory indicators in lung sections. Briefly, both primary antibodies (Abcam Ltd., USA) were incubated with deparaffinized tissue sections before the reagents needed for the ABC reaction were incubated with them (Vectastain ABC-HRP Kit, Vector Laboratories). After that, sections were marked with peroxidase and DAB-chromogen substrate (Sigma), whereupon inspected by a light Olympus microscope. Image J software was used to measure the expression of both immune markers by calculating the percentage area (total number of pixels/field) in 3 fields for each section (total 7 sections) per group.

### Quantitative RT-PCR for TNF-α, IL-1β, IL-10, TGF-β Genes

Using the RNeasy mini extraction kit, total RNA was isolated from the tissue samples in line with the manufacturer’s recommendations (Qiagen). Following the use of DNase I to remove DNA contamination (Fermentas, Lithuania), complementary DNA (cDNA) was produced using a RevertAid First Strand cDNA Synthesis Kit (Thermo Scientific) in accordance with the manufacturer’s instructions. The Rattus Norveicus sequences found in Gen Bank were used to create the primer sets for assessing the mRNA levels of specific genes (Table 1). The primers were created using the primer3 software. Real-time PCR analysis was performed to evaluate the relative expression of the chosen genes using the SYBR Green PCR Master Mix (Thermo scientific Cat number: 4309155). Using the manufacturer’s instructions with the Applied Biosystem’s ABI Prism StepOnePlus Real-Time PCR System [28], the PCR reactions were carried out twice for every sample. The expression levels of the housekeeping gene beta actin were used to normalize the expression levels of interleukin-1 beta (IL-1β), interleukin-10 (IL-10), transforming growth factor-1 beta (TGF1β), and tumor necrosis factor-alpha (TNF-α). The DDCt technique was used to analyze the gene expression data [29].

**Table 1:** The primer sets of the specific genes

	Sense	Antisense	Amplicon	Accession no
*IL-1β*	TTGAGTCTGCACAGTTCCCC	GTCCTGGGGAAGGCATTAGG	161	NM_031512.2
*IL-10*	TCCCTGGGAGAGAAGCTGAA	CCTGCAGTCCAGTAGATGCC	234	NM_012854.2
*TGF-1β*	TACGCCAAAGAAGTCACCCG	GTGAGCACTGAAGCGAAAGC	357	NM_021578.2
*TNF-α*	ACACACGAGACGCTGAAGTA	GGAACAGTCTGGGAAGCTCT	235	NM_012675.3
*ACTB*	CCGCGAGTACAACCTTCTTG	CAGTTGGTGACAATGCCGTG	297	NM_031144.3

**Abbreviations:** *IL-1β* interleukin 1 beta, *IL-10* interleukin 10, *TGF-1β* transforming growth factor 1 beta, *TNF- α* tumor necrosis factor alpha, *ACTB* housekeeping gene beta actin

### Determination of Cobalt and Iron Levels in Lung Tissues

The levels of both cobalt (Co) and iron (Fe) were determined using flame atomic absorption spectroscopy (AAS 5 FL, Carl Zeiss Jena GmbH, Germany) according to the method described by Hassanen et al. [30].

### Statistical Analysis

The parametric data including oxidative stress and gene expression were analyzed by one-way analysis of variance (ANOVA) followed by the post hoc Duncan’s test utilizing the SPSS version 25; *p* values less than 0.05 reflect the statistical significance. Data were described as the mean and standard deviation of the mean (mean ± SD). The nonparametric data including histology lesion rating were presented as median and analyzed by the Kruskal-Wallis *H* test followed by Mann-Whitney *U* test.

## Results

### Characterization of Nanoparticles

**Fig. 1:**
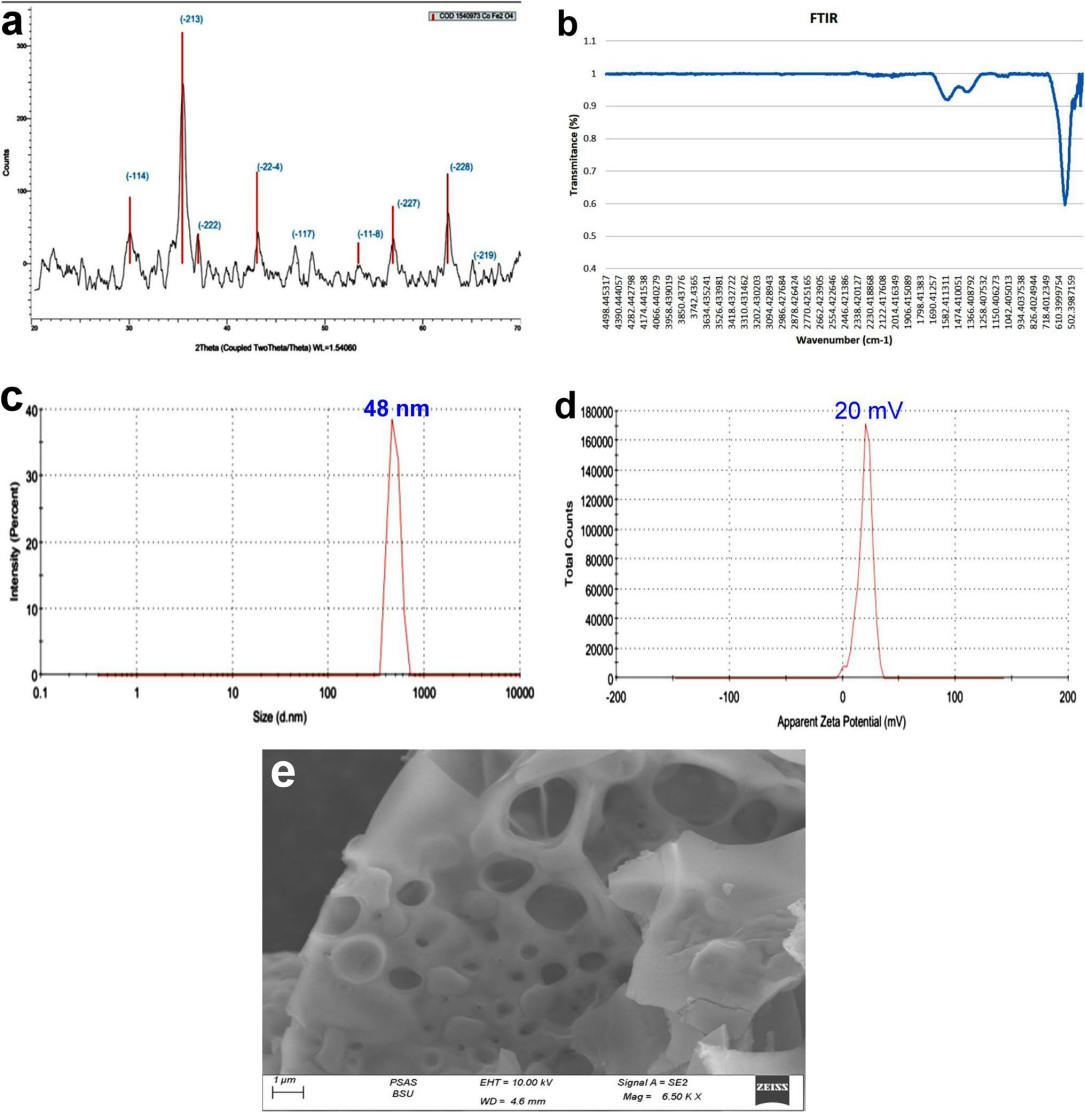
Characterization of CFN, (**a**) X-ray diffraction chart, (**b**) FTIR chart, (**c**) particle size distribution curve, (**d**) zeta potential of CFN, and (**e**) field emission scanning electron microscopic image illustrated the morphology of the prepared nanoparticles

Figure 1a illustrated X-ray diffraction (XRD) chart of the prepared powdered sample without further heat treatment. The data were easily indexed using the JCPDS file COD1540973. All peaks are broad with small intensities which is the fingerprint of nanoscale crystallites. Despite the small size of the nanoparticles, their crystallinity is fairly excellent. The peaks indicated the formation of the Co ferrite in the hexagonal phase adopting the space group R3-m. The crystallite size of nanoparticles was calculated according to Scherrer’s formula and found to be 30 nm. FTIR chart (Fig. 1b) reveals the formation of the ferrite nanoparticles and assures the obtained phase in XRD. The transmittance at 470 and 418 cm^−1^ pointed to the metal oxygen bond (Co–O) in the nanoparticles, while the band appeared at 555 cm^−1^ was ascribed to Fe–O bond [31]. From the DLS data presented in Fig. 1c, the hydrodynamic diameter is peaked around 48.4 nm with a poly-dispersibility index PDI=1. Moreover, Fig. 1d illustrates the values of the measured zeta potential of the magnetic nanoparticles under investigation. The data showed positive large values of 20 mV which means that the particles possess positive surface charges. Additionally, the large value is distinguished and applicable as the stability herein is high in the solution. The morphology of the prepared nanoparticles is imaged and represented in Fig. 1e. The SEM photo demonstrates large grains with rocky-like morphology. Many types and different shapes of pores are depicted assuring the porous like nature of the fine powder. The nanoparticles under study will be promising candidates owing to their large surface area and closed pores of different sizes.

### Oxidative Stress Evaluations

**Fig. 2:**
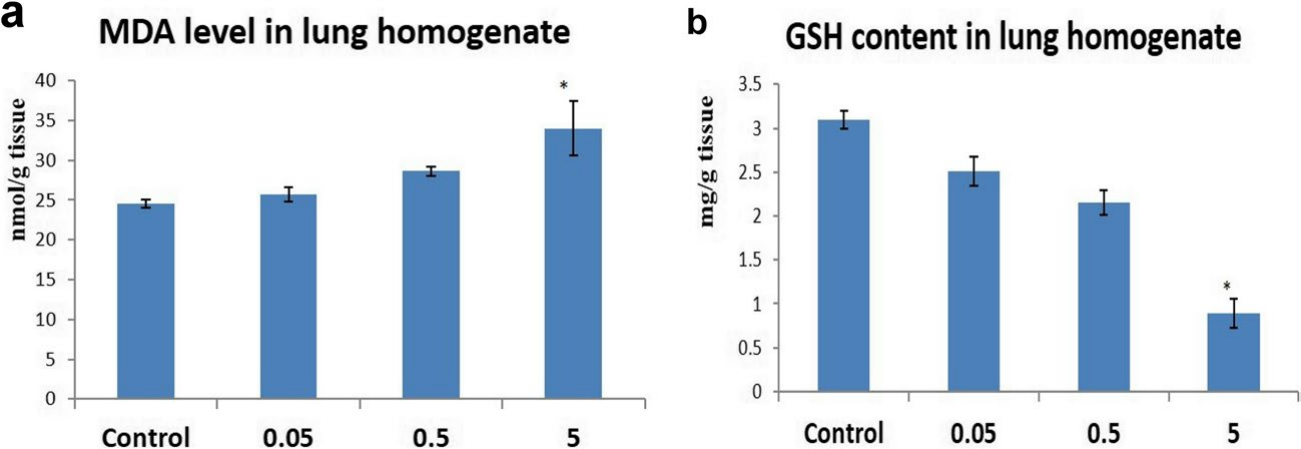
Effects of the administration of different dose levels of CFN on the pulmonary levels of oxidant/antioxidant biomarkers. (**a**) MDA levels and (**b**) GSH activity. Values are presented as mean ± SD (*n* = 7 rats/group). * *p* < 0.05 vs control group

The group receiving the highest dose level showed a significant decrease in GSH content and a significant increase in MDA level in the lung compared to the control group. Additionally, exposure to low and middle doses of CFN (0.05 and 0.5) resulted in a non-significant change in GSH and MDA levels compared to the control group (Fig. 2).

### Histopathological Examination

**Fig. 3:**
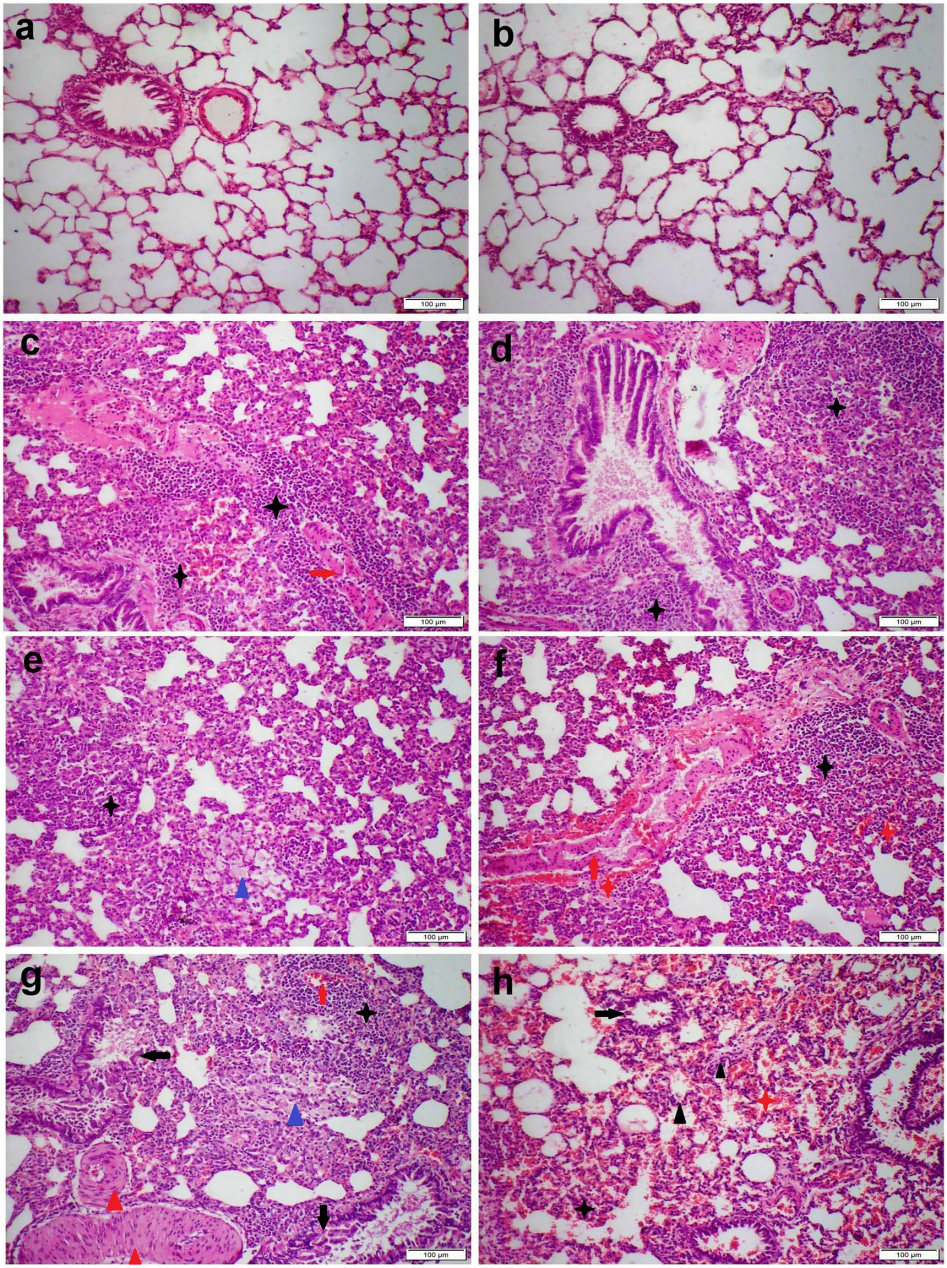
Microscopic images of H&E stained lung sections representing different treatment groups as follows: (**a**) control group with normal microscopic appearance, (**b**) 0.05 mg CFN group showed mild septal thickening, (**c–e**) 0.5 mg CFN group and (**f–h**) 5 mg CFN group showed severe histopathological alterations. Note: inflammatory cell infiltration (black stars), vasculitis (red arrows), hemorrhage (red stars), edema (blue triangles), concentric laminated muscular hyperplasia (blue triangles), bronchial and bronchiolar epithelial necrosis (black arrows)

Pulmonary tissue of the control group showed the normal histological structure of the bronchi, bronchioles, alveoli, and interstitium (Fig. 3a). Likewise, the group receiving 0.05 mg magnetic NPs showed a normal microscopic appearance except for alveolar capillary congestion and mild septal thickening (Fig. 3b). On the other hand, group receiving 0.5 mg magnetic NPs showed moderate interstitial pneumonia. There was multifocal lymphoplasmacytic cell infiltration in the interstitial tissue along with diffuse septal thickening (Fig. 3c). Some bronchi and bronchioles showed moderate desquamation in their epithelial linings with intraluminal mononuclear inflammatory cell aggregation (Fig. 3d). Most pulmonary arteries and arterioles showed concentric laminated muscular hyperplasia (Fig. 3e). Group receiving 5 mg NPs showed severe interstitial pneumonia accompanied with diffuse alveolar damage (Fig. 3f). The majority of blood vessels exhibit vasculitis along with perivascular lymphocytic cuffing (Fig. 3g). Most bronchial and bronchiolar epithelial cells showed necrosis of its epithelium with intraluminal inflammatory cell aggregation (Fig. 3h).

All NPs receiving group showed a significant increase in the histological lesion scoring compared with the control group, but the highest score in all parameters was recorded in the group receiving 5 mg NPs (Table 2).

**Table 2:** The microscopic lesion scoring in different experimental groups

	Control	0.05	0.5	5
Congestion	1 ^a^	2 ^b^	4 ^c^	5 ^d^
Vasculitis	1 ^a^	1 ^a^	3 ^b^	5 ^c^
I.S. inflammation	1 ^a^	2 ^b^	4 ^c^	5 ^d^
I.S. hemorrhage	1 ^a^	1 ^a^	1 ^a^	5 ^b^
I.S. edema	1 ^a^	1 ^a^	3 ^b^	5 ^c^
Alveolar damage	1 ^a^	1 ^a^	3 ^b^	5 ^c^
Bronchial damage	1 ^a^	1 ^a^	3 ^b^	5 ^c^

Values are presented as median (*n* = 7 rats/group). Different superscripts mean significant difference at *p* < 0.05

### Immunohistochemical Staining

**Fig. 4:**
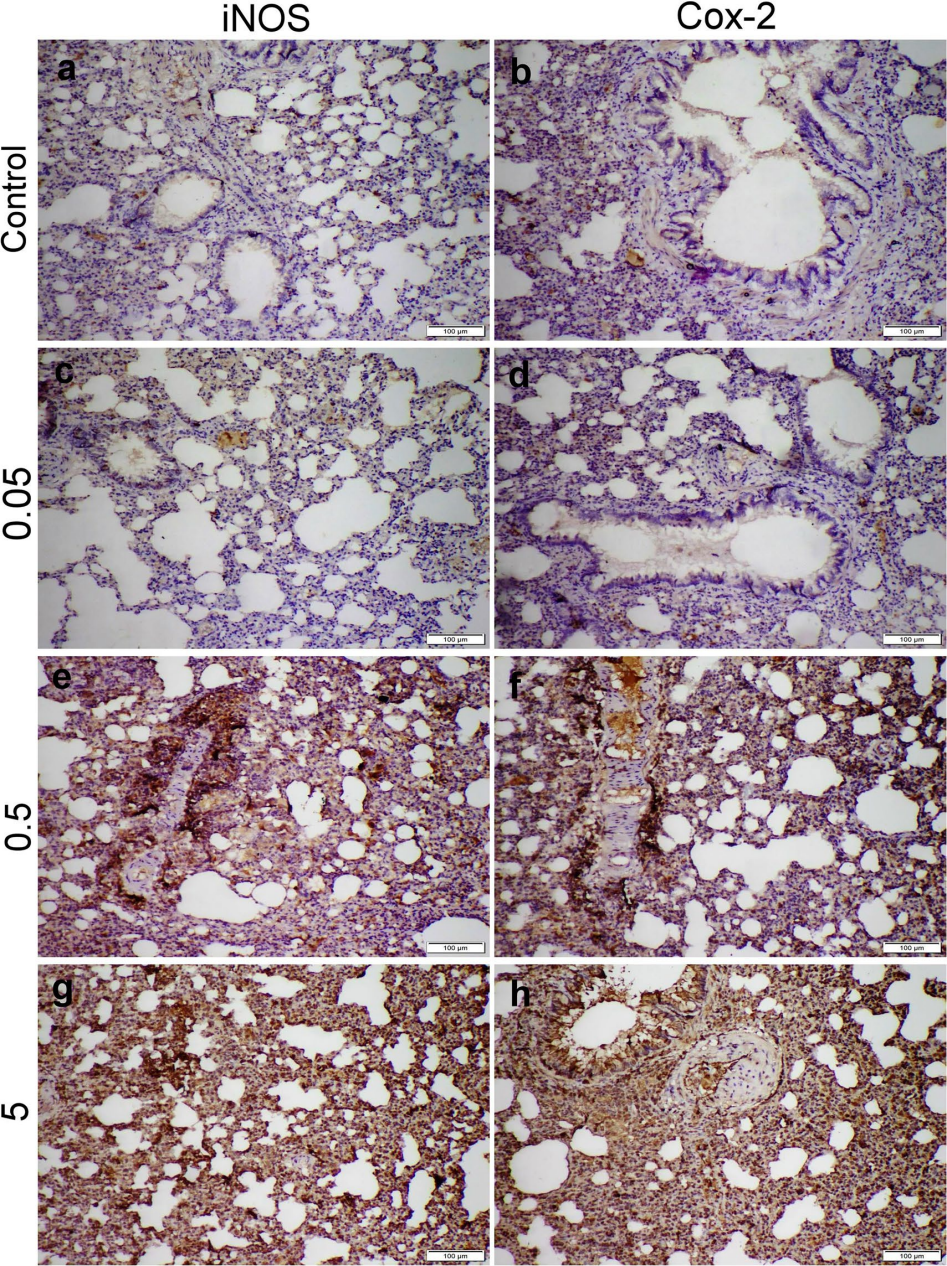
Microscopic images representing iNOS and Cox-2 immunostaining expression in different treatment groups. (**a, b**) Control group with negative immunoexpression, (**c, d**) 0.05 mg CFN group with mild immunoexpression, (**e, f**) 0.5 mg CFN group, and (**g, h**) 5 mg CFN group, both showed strong immunopositivity of both immune markers

The lung sections obtained from the control group showed negative iNOS and Cox-2 protein expressions. Otherwise, all NPs given groups demonstrated dose-dependent increase in both immune markers. The highest immunohistochemical reaction were recorded in 5 mg NPs receiving group followed by those receiving 0.5 mg NPs, whereas 0.05 mg NPs receiving group displayed negative to weak positive immune reactions (Fig. 4).

### Quantitative RT-PCR for TNF-α, IL-1β, IL-10, TGF-β Genes

**Fig. 5:**
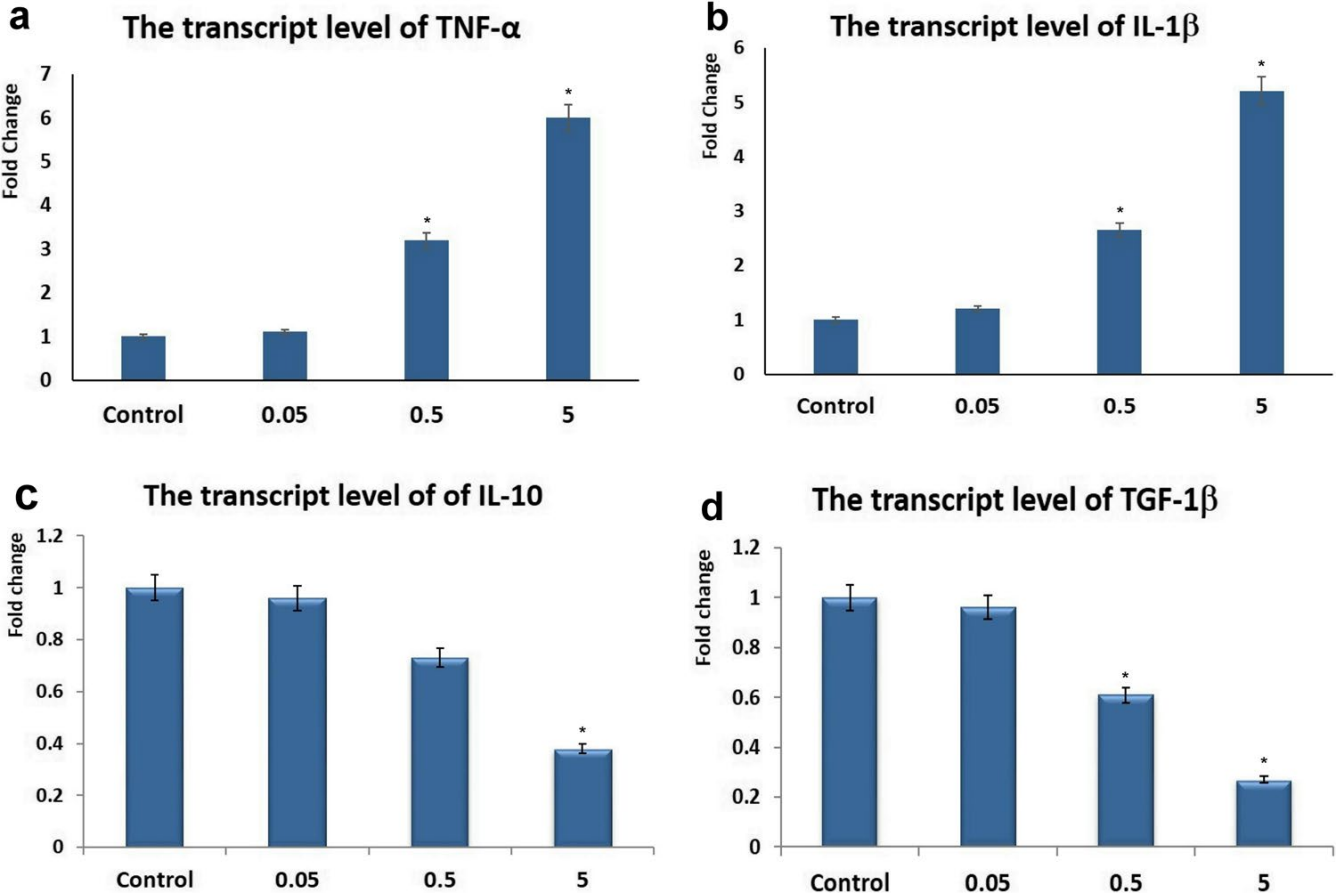
Effects of the administration of different dose levels of CFN on the pulmonary levels of some inflammatory genes. (**a**) TNF-α, (**b**) IL-1β, (**c**) IL-10, (**d**) TGF-1β. Values are presented as mean ± SD (*n* = 7 rats/group). * *p* < 0.05 vs control group

The expression levels of the m-RNA proinflammatory genes (TNF-α and IL-1β), as well as the anti-inflammatory (IL-10 and TGF-1β), were assessed in the lung tissue of different experimental groups. The transcript level of TNF-α and IL-1β showed significant upregulation in the middle and high doses of NPs. On the other hand, the transcript level of IL-10 and TGF-1β showed downregulation in the middle and high doses group. Furthermore, the transcript level of the abovementioned genes did not show any significant difference in the low dose group compared with the control group (Fig. 5).

### Cobalt and Iron Levels in Lung Tissues

**Fig. 6:**
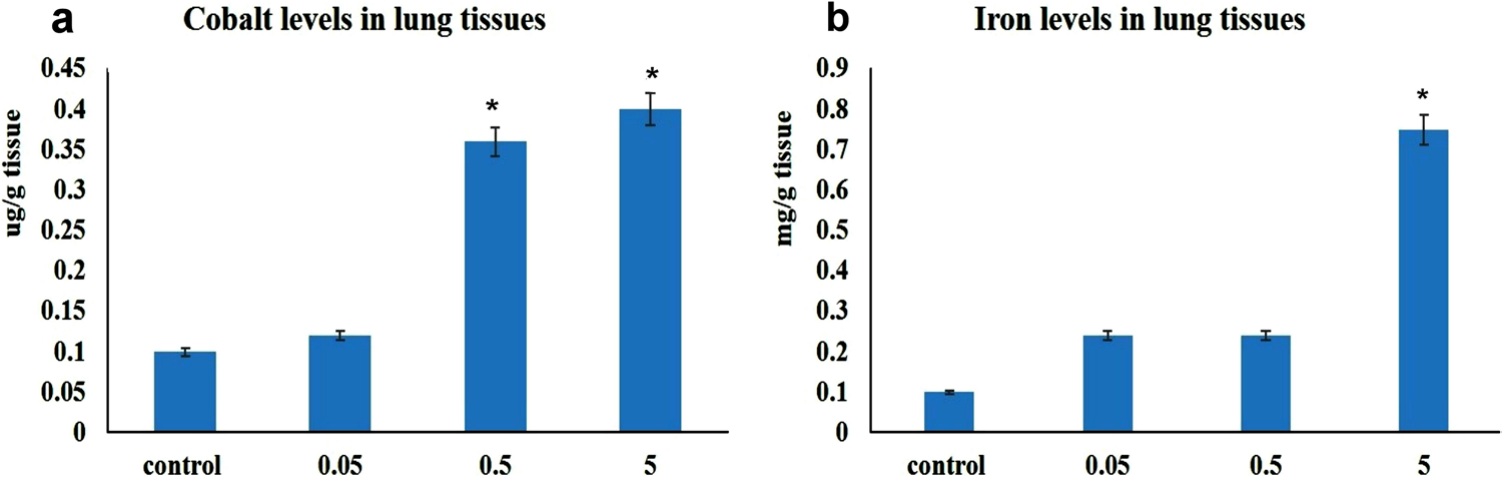
Graph demonstrated (**a**) cobalt (Co) and (**b**) iron (Fe) levels in lungs of different groups. Values are presented as mean ± SD (*n* = 7 rats/group). * *p* < 0.05 vs control group

The highest levels of both cobalt and iron were recorded in 5 mg CFN receiving group. Moreover, a significant raise in cobalt level was recorded in the lungs obtained from 0.5 mg CFN receiving group compared with the control group. On the other hand, 0.05 mg NPs receiving group did not show any significant difference in both cobalt and iron levels compared with the control group (Fig. 6).

## Discussion

CFN are among the most commonly utilized magnetic nanoparticles (MNPs) in medicine. For example, they are applied in magnetic resonance imaging (MRI) and cancer treatment [32], medications delivery [33], diagnostics [34], and electronic devices [35]. CFN are frequently used in various applications, which leads to a significant environmental disposal and a worrisome scenario to both humans and animals [36]. Co^2+^ and Fe^3+^ ions were released into the environment as a result of the biodegradation of CFN. Studying the potentially harmful consequences of CFN and the subsequent release of their ions into the biological system is therefore urgently necessary. Furthermore, the route of administration is essential in estimating MNP toxicity as different exposure routes produced various toxicological outcomes. Our goal in this study is to investigate the possible mechanism of CFN- inducing pulmonary toxicity in rats after repeated oral intake especially from the molecular and histopathological insights. Regarding CFN characterization, XRD peaks are characteristic for CFN [31]. FTIR spectra showed characteristic peaks at 470 and 418 cm^−1^ pointed to the metal oxygen bond (Co–O) in the nanoparticles, while the peak at 555 cm^−1^ represented Fe–O bond [3, 31].

In the present study, we found that CFN induced dose-dependent rise in MDA levels and decline in the GSH contents in the pulmonary homogenates confirming the oxidative stress injury induced by CFN. Previous studies proved the ability of both Co^2+^ and Fe^3+^ ions to induce reactive oxygen species (ROS)-mediated oxidative stress, cytotoxicity, genotoxicity, inflammations, and apoptosis [37–41]. By promoting the creation of ROS, NPs can cause oxidative stress and cell death in living organisms [42, 43]. The result of the oxidative stress reflects on the microscopic picture of lung tissue that demonstrates dose-dependent pulmonary inflammation and cellular damage. In previous investigations, few studies had reported the deleterious effects of CFN on the lungs. For example, inhalation of CFN induced direct damage and increased contractility in smooth muscles of the lungs in a guinea pig experimental model [44]. To the best of our knowledge, our study is the only to investigate the effects of CFN on lungs when administered via ingestion route. Magnetic NPs in general are targeting liver and spleen as the major organs when ingested or administered intravenously into the body, which causes numerous inflammations and cell injuries in these organs [45]. However, other organs such as kidney, endocrine glands, heart, and lungs should be studied as well. CFN could target the lung tissue since this organ possesses alveolar macrophages, which have a cleaning function and are important in phagocytosing nano- and micromaterials from the bloodstream. Additionally, the lungs are rich in important oxidase enzymes that are essential for the detoxification of a variety of toxins. Our histopathological findings were confirmed the previous Orel’s findings who found that CFN often cause apoptosis and necrosis at higher concentrations, but they typically cause cellular proliferation at lower concentrations [46].

The aggregation and/or mechanical injury of the CFN may contribute to their lung toxicity. Because of its greater affinity for membranes and greater cytotoxicity, agglomerates of Fe_2_O_3_ are more harmful to lipid membranes [47, 48]. The main causes of NPs-induced lung toxicity were also the adhesion of aggregates, internalization of NPs, and released ions [49]. We found a significant elevation of both cobalt and iron levels in lungs obtained from 5 mg NPs receiving group compared with the other groups. In contrast to the iron, the cobalt levels only increased in the lung obtained from 0.5 mg NPs receiving groups. CFN are subjected to break down inside the body to iron and cobalt; while cobalt could be deposited in different organs, iron is utilized for metabolic functions [50]. The cumulative impact of internalization and aggregation of either CFN or its leashed ions (Fe^3+^ and Co^2+^) is discovered to be the reason for increasing the value of lipid peroxidation by increasing the CFN concentration. Both NPs and their released ions encouraged the creation of pores by lipid peroxidation and magnetolysis [32], making them more permeable and less selective [51]. Additionally, its buildup in some organs promoted the disruption of particular processes in other cellular compartments. Because of the oxidative degradation of long chains of amino acids, the expression of several proteins increased [52].

It is well known that the oxidative stress initiates several pathological conditions including inflammatory reactions. Various phagocytic cells, such as neutrophils and macrophages, were drawn to the pulmonary tissue as a result of ROS-mediated endothelium activation and overexpression of adhesion molecules leading to the synthesis of inflammatory cytokines and chemokines [53]. In addition to proteolytic enzymes, ROS, cationic proteins, lipid mediators, and extra-inflammatory cytokines are among the cytotoxic substances that are also produced by these cells [54]. Attracting more inflammatory cells creates more cytotoxic mediators and supports a negative cycle that eventually results in respiratory failure due to severe damage to the alveolo-capillary membrane ends. These findings suggest our results about the dose-dependent increase in both iNOS and Cox-2 protein expressions and upregulation of TNF*α* and IL-1*β* along with downregulation of IL-10 and TGF-1β. TGF-1β and IL-10, the inhibitory cytokines, are well-known for their anti-inflammatory activities [55]. TGF-1β is produced by the active T-lymphocyte, afterward; it inhibits the T-cell proliferation as a negative feedback role. The previous report explained that TGF-1β suppresses the mRNA expression level of the IL-2, the cytokine responsible for T-cell proliferation [56]. IL-10 exerts its anti-inflammatory activity by suppressing the pro-inflammatory cytokines (TNF-α, IL-1β, and IL-12) and inhibiting the macrophages’ functions [57]. In the current study, the groups receiving the middle and high doses of CFN showed a significant down-regulation in the expression levels of TGF-1β and IL-10. This decrease explains the pulmonary inflammation revealed by the histopathological examination.

In our study, histopathological lesions were dose-dependent and mainly of inflammatory nature. Inflammation results in the overexpression of various proinflammatory enzymes and reactive species like superoxide and nitric oxide (•NO) radicals [58]. Inducible nitric oxide synthase (iNOS), an enzyme that is up-regulated during the inflammatory process, produces •NO from oxygen and L-arginine [59, 60]. Prostaglandins (PGs) are yet another crucial mediator that is overexpressed during inflammation. Cyclooxygenase (Cox) activity on arachidonic acid, followed by PG synthase activity, produces PGs, which are bioactive signaling molecules. Cox-2 is significantly elevated in response to numerous inflammatory disorders, like iNOS [61]. Thus, we observed dose-dependent immunoreactivity to iNOS and Cox-2 in CFN receiving group with the strongest reaction in 5 mg group. Important signaling chemicals like PGI2, thromboxane A2, PGE2, PGD2, and PGF_2α_ can be produced by PG synthases [62]. During inflammation, several pro-inflammatory cytokines as IL-1β and TNF-α regulate the upregulation of iNOS and Cox-2 [63]. Some researchers discovered that the inflammatory cytokines may directly control the local inflammatory response that develops surrounding injured sites [64, 65]. IL-1 and TNFα signaling are also reportedly engaged in the processes of tissue regeneration following tissue disruption brought on by a variety of traumas [66–68].

## Conclusion

In this study, we found that the pulmonary toxicity induced by CFN in rats not only related to the internalization of NPs itself, but also related to the aggregation of its leashed components (cobalt and iron) in the lung tissues. Our results showed that both the high and middle doses of CFN prompted injurious influences on the rats’ lungs. The toxicity was exhibited by an elevation in MDA and a decline in catalase enzyme. Moreover, CFN exerted interstitial pulmonary inflammation along with the bronchial and alveolar damages, which were confirmed by immunohistochemistry, demonstrating the increased iNOS and Cox-2 protein expression. Likewise, the transcript levels of the studied genes revealed up-regulation of the pro-inflammatory genes and downregulation of the anti-inflammatory genes.
